# Assessing the prevalence of mycoplasma contamination in cell culture via a survey of NCBI's RNA-seq archive

**DOI:** 10.1093/nar/gkv136

**Published:** 2015-02-24

**Authors:** Anthony O. Olarerin-George, John B. Hogenesch

**Affiliations:** Department of Systems Pharmacology and Translational Therapeutics, Perelman School of Medicine at the University of Pennsylvania, Philadelphia, PA 19104, USA

## Abstract

Mycoplasmas are notorious contaminants of cell culture and can have profound effects on host cell biology by depriving cells of nutrients and inducing global changes in gene expression. Over the last two decades, sentinel testing has revealed wide-ranging contamination rates in mammalian culture. To obtain an unbiased assessment from hundreds of labs, we analyzed sequence data from 9395 rodent and primate samples from 884 series in the NCBI Sequence Read Archive. We found 11% of these series were contaminated (defined as ≥100 reads/million mapping to mycoplasma in one or more samples). Ninety percent of mycoplasma-mapped reads aligned to ribosomal RNA. This was unexpected given 37% of contaminated series used poly(A)-selection for mRNA enrichment. Lastly, we examined the relationship between mycoplasma contamination and host gene expression in a single cell RNA-seq dataset and found 61 host genes (*P* < 0.001) were significantly associated with mycoplasma-mapped read counts. In all, this study suggests mycoplasma contamination is still prevalent today and poses substantial risk to research quality.

## INTRODUCTION

Mycoplasmas are small parasitic bacteria of the class mollicutes. There are over 180 species infecting a wide range of hosts. Mycoplasmas are common to the human respiratory and urogenital tracts (1). Some species are pathogenic. For example, *Mycoplasma pneumoniae* causes atypical pneumonia (2). Also, *Mycoplasma genitalium* infection is linked to pelvic inflammatory disease (3).

In addition to their impact on human health, mycoplasmas are widespread contaminants of cell culture. In 1956, researchers from Johns Hopkins reported mycoplasma contamination of HeLa cells used in their lab (4). By the early 1990s, the US Food and Drug Administration had tested over 20 000 cell cultures and found 15% contaminated with mycoplasma (5). A study in 1991 from Argentina found 70% of the 200 samples tested were contaminated (6). More recently, a 2002 report by the Deutsche Sammlung von Mikroorganismen und Zellkulturen (DSMZ) in Germany found 28% of the 440 cell lines tested (mostly leukemia-lymphoma) were contaminated (7). Hence, mycoplasma contamination in cell culture is a long-standing and persistent problem.

Preventing mycoplasma contamination is difficult. For one, mycoplasma cells are small (0.3–0.8 µM in diameter) and pleomorphic (8), allowing them to pass through standard filtration membranes. Secondly, mycoplasmas lack cell walls. This makes them impervious to cell culture antibiotics that inhibit cell wall synthesis, such as penicillin. Effective antibiotics do exist, however, their continuous use in cell culture is not recommended due to possible cytotoxicity. Third, mycoplasmas are able to reach high concentrations in the media of infected cells without noticeable turbidity (9). This makes detection via visual inspection difficult. Lastly, while the primary source of mycoplasma contamination is likely other cell cultures, mycoplasmas from lab personnel are a potential source as well (7). Hence, the only reliable ways to minimize contamination are to test routinely (and frequently), practice safe cell culture techniques and to obtain cells from reputable sources such as the American Type Culture Collection (ATCC).

Mycoplasmas have one of the smallest prokaryotic genomes (about 0.6 Mb). This comes at a price, as mycoplasmas lack key genes essential for the synthesis of macromolecule precursors and energy metabolism (10). As a result, mycoplasmas alter and depend on host cell biology for survival (11,12). For example, *Mycoplasma orale* can compete for arginine in culture media, impacting host cell growth (13). Also, *Mycoplasma hyorhinis* endonucleases can degrade host cell DNA, providing DNA precursors for the parasite (14). Further, mycoplasma infection can disregulate hundreds of host genes (15). It is therefore imperative that cultured cells are free of mycoplasma contamination to ensure the interpretability, reproducibility and reliability of results obtained from their use.

In this study we sought to evaluate the prevalence of mycoplasma contamination in cell culture today. High-throughput RNA-sequencing data is growing at an exponential rate and is providing an unprecedented view of the constituency of RNA molecules in a sample. We posited that mycoplasma sequences in RNA-Seq data from primate and rodent specimens would be indicative of contamination. Hence, we surveyed RNA-Seq data from archives at NCBI for mycoplasma sequences. We also evaluated the relationship between mycoplasma contamination and host gene expression in a Burkitt's lymphoma cell line.

## MATERIALS AND METHODS

All Perl scripts used in this study are available as supplemental files. Directions for their use are located in the README file. The basic local alignment search tool (BLAST) analysis was performed through Amazon Web Services (8-core, 70 GB of RAM). All other analyses were done on the high-performance computing cluster of the Penn Genomes Frontier Institute; a shared resource of 1000 compute cores and at least 8 GB of memory per core.

### Obtaining sequence files

Gene expression omnibus (GEO) series IDs and descriptions for all RNA-seq experiments were obtained through the GEO DataSets Advanced Search Builder (http://www.ncbi.nlm.nih.gov/gds/advanced). Results were limited on the query parameter ‘dataset type’ for ‘expression profiling by high-throughput sequencing’ and ‘non-coding RNA profiling by high-throughput sequencing’. The results were further filtered to include only primates (*Homo sapiens, Pan troglodytes, Macaca mulatta, Macaca fascicularis, Pan paniscus, Gorilla gorilla*) and rodents (*Mus musculus, Rattus novergicus*). Simple Omnibus Format in Text (SOFT) files for all the GEO series were downloaded. From these files, the sample IDs were obtained and used to download the corresponding raw sequences using the fastq-dump utility of the SRA toolkit (http://eutils.ncbi.nih.gov/Traces/sra/?view=software).

### Identifying poly(A)-selected and cultured samples from sample descriptions

SOFT files were parsed and searched for keywords under certain headings. For example, poly(A) selection was assumed if the library source or extraction protocols contained the keywords ‘polyA’ or ‘oligo-dT’ and similar variants. If a sample did not contain these keywords we labeled it ‘other’. This group was heterogeneous containing rRNA depleted, size selected and even poly(A)-selected samples. Unfortunately, for these samples, the exact designation could not be determined programmatically from the GEO description alone. A sample was considered cultured if its description contained the keywords ‘cell line’, ‘fibroblast’, ‘MEFs’, ‘ESC’, ‘immortalized’, ‘DMEM’, ‘RPMI’, ‘Ham's’, ‘McCoy’, ‘cultured’, ‘passaged’, ‘propagated’, ‘grown’ and similar variants of these keywords. If a sample did not contain these keywords we considered it non-cultured. We confirmed the accuracy of the program by manually annotating 100 randomly selected samples and comparing the results. The program correctly identified 96% of cultured samples, 85% of non-cultured samples and 90% of poly(A)-selected samples.

### Mapping sequence reads to mycoplasma genomes

The mycoplasma genomes used in this study were downloaded from NCBI genomes: *Mycoplasma hominis* ATCC 23114 (NC_013511.1), *M. hyorhinis* MCLD (NC_017519.1), *Mycoplasma fermentans* M64 (NC_014921.1) and *Acholeplasma laidlawii* PG-8A (NC_010163.1). RNA-seq reads were mapped to each of these genomes separately with Bowtie 1 using the default parameters (16). If a read was paired-end, only the first read was used in the analysis.

### Filtering non-mycoplasma-specific sequences from Bowtie results

All unique Bowtie-mapped reads were aligned to NCBI's nucleotide (nt) database using standalone BLAST+ (http://www.ncbi.nlm.nih.gov/books/NBK1763/). The following parameters were used: -db nt -num_threads 8 -outfmt ‘7 std sscinames’ -max_target_seqs 100. A Bowtie-mapped read was considered unique to mycoplasma if: (i) the scientific name matched ‘Mycoplasma’ or ‘*A. laidlawii*’ in the BLAST output and (ii) the entry from (i) was the best BLAST hit (i.e. with the lowest *E*-value).

### Determining mycoplasma effect on host gene expression

The complete sequences for the seven samples comprising series GSE49321 were downloaded with the SRA toolkit. GSE49321 is a single-cell RNA-seq dataset of the Burkitt's lymphoma cell line DG-75. The sequences were aligned to the human genome (hg19) using STAR (17) with the following optional parameters: –runMode alignReads –runThreadN 8. Read counts for each gene were determined using HTSeq with the following optional parameters: -s no -m intersection-nonempty. Gene expression (counts) was modeled as a function of mycoplasma-mapped reads using DESeq2 (18).

## RESULTS

### Acquisition and characterization of RNA-seq data

RNA-seq is a high-throughput method for determining the sequence (and quantity) of RNA molecules. Most peer-reviewed journals require deposition of RNA-seq data in a publicly accessible database such as the NCBI Sequence Read Archive (SRA) as a condition of publication. We assessed the feasibility of working with the large volume of sequencing data in SRA. At the start of this study (April 2013), there were 884 series (or projects) of type ‘expression profiling by high throughput sequencing’ or ‘non-coding RNA profiling by high throughput sequencing’ from primates and rodents (Supplementary File S1). These were comprised of 9395 samples with downloadable sequence files. To make the analysis manageable, we downloaded just the first million reads of each of the samples using NCBI's SRA toolkit. For samples with paired-end reads, we assessed only the first read. The resulting dataset contained 6.4 billion reads and filled 2TB of disk space.

### Mapping of reads to mycoplasma

**Figure 1.**
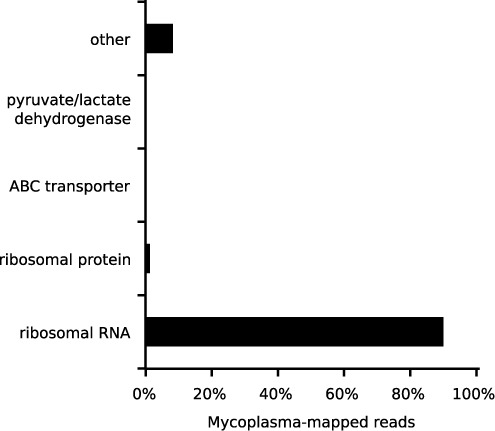
Gene breakdown of mycoplasma-mapped reads. RNA-seq reads were aligned to four mycoplasma genomes using bowtie. Non-specific reads were filtered with BLAST. Of the resulting 472 219 mycoplasma-mapped reads, 90% mapped to mycoplasma ribosomal RNA.

We mapped the reads to mycoplasma in two steps. First, we aligned the reads to four complete mycoplasma genomes (*M. hominis* ATCC 23114, *M. hyorhinis* MCLD, *M. fermentans* M64 and *A. laidlawii* PG-8A) with Bowtie (16). These species were chosen because they are frequently found in contaminated samples and had complete genomes in NCBI. Less than 2% of the 6.4 billion reads (112 547 232) mapped to one or more of the mycoplasma species. This corresponded to 1 280 951 unique sequence reads. Next, we eliminated non-specific Bowtie aligned reads. These were reads that mapped to sequences from other sources as well as or better than they did mycoplasma. For example, reads that mapped to the putative host species or to other bacteria were eliminated. To accomplish this, we aligned the unique Bowtie-mapped reads to the NCBI nucleotide collection (nt) database with standalone BLAST+ (19). The nt database is a comprehensive collection of about 20 million DNA sequences from species spanning the phylogeny of life and viruses. These sequences include entries from Refseq, GenBank, EMBL and DDBJ. Of the 1 280 951 unique Bowtie aligned reads, 474 219 (37%) aligned best to mycoplasma as assessed by BLAST *E*-values. Ninety percent of all BLAST-confirmed reads aligned to ribosomal sequences (Figure 1). Lastly, we tested for bias in selecting the first million reads of each sample for this study. We downloaded the complete sequence data for 40 samples with varying amounts of mycoplasma-mapped reads (see Supplementary Table S1). The number of reads per sample ranged from 1.4 to 93 million. The median was 21 million. We compared the fraction of mycoplasma-mapped reads per million for the first 1 million reads versus the entire dataset. The R^2^ value was 0.99 suggesting no bias (Supplementary Figure S1).

### Mycoplasma contamination in cultured versus non-cultured cells

**Figure 2.**
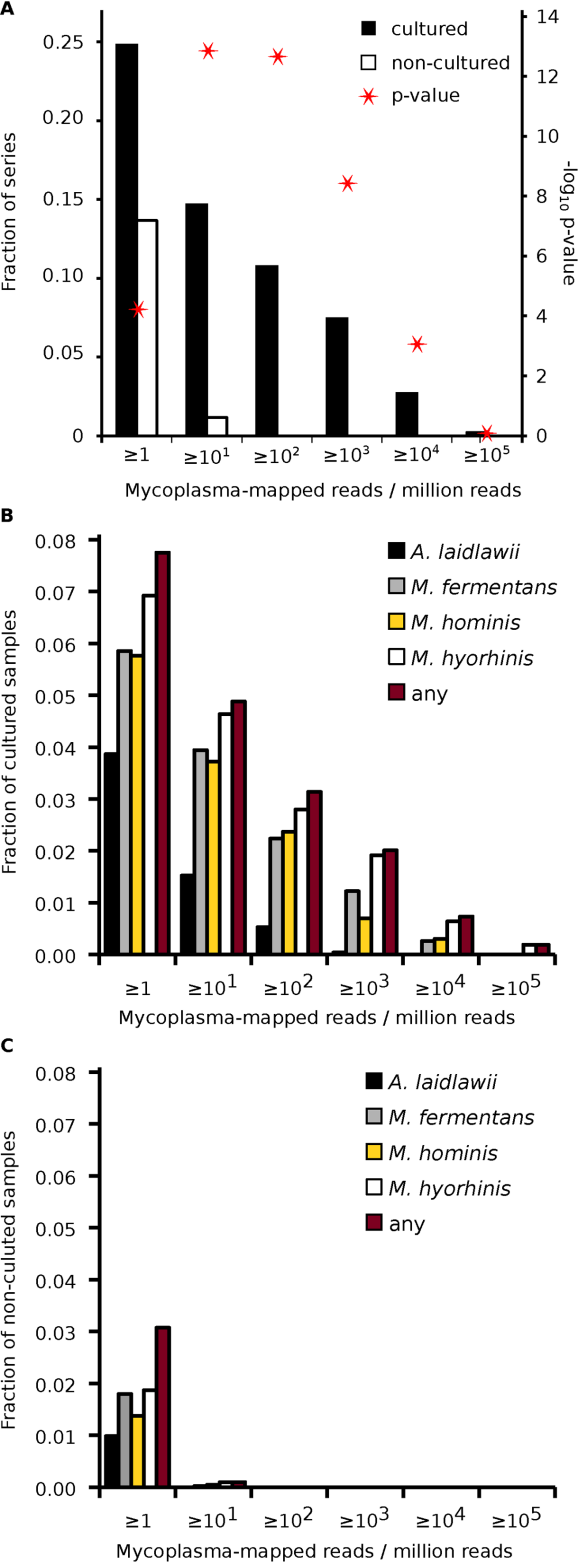
Mycoplasma contamination in cultured versus non-cultured samples and series. (**A**) Fraction of series that are contaminated (containing cultured samples or not) at various cutoffs of mycoplasma-mapped reads per million (column graphs; primary y-axis). Red stars indicate the *P*-values of the respective comparisons (secondary y-axis; Fisher's exact test). Fraction of contaminated (**B**) cultured or (**C**) non-cultured samples for various cutoffs of mycoplasma-mapped reads per million, broken down by the indicated mycoplasma species.

Mycoplasma contamination is predominantly found in cultured samples (e.g. cell lines) and not in non-cultured samples (e.g. tissues) (20,21). Hence, we posited that non-cultured samples in our study would be under-represented for mycoplasma contamination and provide a baseline for the expected number of mycoplasma-mapped reads in a given sample (i.e. serve as a negative control). We mined the text of the sample descriptions from NCBI's GEO for keywords indicating if the samples were cultured or not (see ‘Materials and Methods’ section). We estimated that 5328 (57%) of the samples were cultured and 4067 (43%) were not (Supplementary Table S2). In series with cultured samples, 11% (52/484) had at least one sample with 100 or more reads per million (RPM) that mapped to mycoplasma (see full distribution in Figure 2A). In contrast, none of the 344 series with non-cultured samples had 100 or more RPM that mapped to mycoplasma (Figure 2A). Further, more samples had reads mapping to *M. hyorhinis*, than the other tested mycoplasma species (Figure 2B and C). Fewer samples had reads mapping to *A. laidlawii*. Surprisingly, for 39 samples (all cultured), more than 10^4^ RPM (i.e >1% of reads) mapped to one or more of the mycoplasma species (Figure 2A, Supplementary Table S2).

### Mycoplasma contamination in poly(A)-selected samples

Polyadenylation marks transcripts for degradation in bacteria (22). Hence, we hypothesized that poly(A)-selected samples would be under-represented for mycoplasma contamination. Also, given that 90% of the mycoplasma-mapped reads were from ribosomal RNA (Figure 1), we hypothesized that poly(A)-selected samples would appear void of mycoplasma contamination. We mined the text of the sample descriptions from NCBI's GEO for keywords indicating if the samples were poly(A)-selected (see ‘Materials and Methods’ section). Surprisingly, we found that 37% of contaminated series (i.e. containing one or more samples with 100 or more mycoplasma mapped RPM) were poly(A)-selected (Supplementary Table S2).

### Effect of mycoplasma contamination on host gene expression

**Figure 3.**
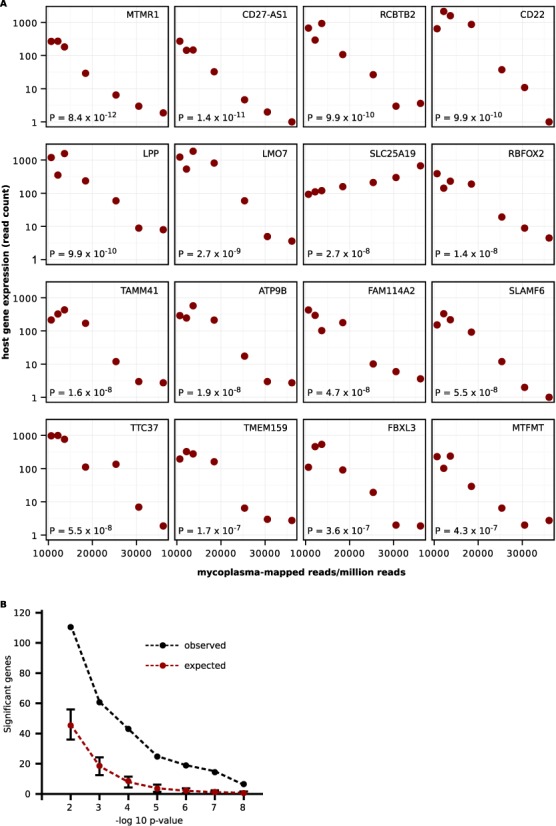
Association between host gene expression and mycoplasma-mapped reads from single-cell RNA-seq. (**A**) Scatter plots of mycoplasma-mapped reads per million and host cell gene expression in single cell (DG-75) RNA-seq. Gene symbols are indicated in the upper right corner of the respective plots. *P*-values from the association test are indicated in the bottom left. (**B**) To assess the likelihood of obtaining significant genes by chance, the mycoplasma counts were permuted 1000 times. The analysis was repeated with each permutation. The expected number of significant genes is plotted in red. The observed number of significant genes is in black. Error bars are standard deviations.

Next, we wanted to determine the effect of mycoplasma contamination on host gene expression. To do this, we searched for series that contained comparable contaminated and non-contaminated samples. This was rare as mycoplasma contamination in one sample typically guaranteed contamination of all samples of the same cell line/type in the series. Hence, we instead focused on contaminated series with large numbers of replicate samples and varied amounts of mycoplasma-mapped reads. Our goal was to identify host genes whose expression levels were statistically associated with the number of mycoplasma-mapped reads. We found one such series (GSE49321) containing seven contaminated samples from the Burkitt's lymphoma cell line DG-75. Interestingly, each sample represented expression data from a single cell. The number of mycoplasma-mapped reads ranged from about 10 000 to 36 000 RPM (Figure 3A). We downloaded the full RNA-seq datasets for these samples, aligned them to the human reference genome using STAR (17) and obtained read counts for genes using HTSeq (23). We then used DESeq2 (18) to generate generalized linear models of host gene expression as a function of mycoplasma-mapped reads. Sixty-one genes were statistically associated with mycoplasma-mapped read counts (Wald test, Benjamini-Hochberg adjusted *P* < 0.001; Supplementary Table S3). We found no significant enrichment in gene annotations using DAVID (24). The top 16 genes are shown in Figure 3A.

The number of mycoplasma-mapped reads across the seven samples may have correlated with the expression of the 61 disregulated genes by chance. With almost 8000 genes this was very plausible. To address this we permuted the expression values for each gene across the seven samples. We then performed the original analysis that gave us 61 significant genes (i.e. the generalized linear models). We repeated this 1000 times. On average, only 19 genes were differentially regulated (hence a 3.2-fold enrichment in the observed set). In fact, the number of significant genes was consistently less than that of the observed set for various *P*-value cutoffs examined (*P* < 0.002, Figure 3B).

### Publication of contaminated studies

Lastly, we wanted to know if the contaminated series were associated with published manuscripts and if so what impact these publications have likely had on their field of study (as assessed by article citations). In Table 1 we list the 20 series with the highest number of mycoplasma-mapped reads, the journals in which they were published (if applicable) and the number of citations the publications have received. Series GSE49321, used in the previous section to determine the effect of mycoplasma contamination on host gene expression, was sixth on the list. All but two series are associated with publications. These include top peer-reviewed journals such as Nature, Cell, Proceedings of the National Academy of Sciences, Genome Research, Genes and Development, RNA and this journal, Nucleic Acids Research. These articles have been generally well cited with half of them receiving at least 51 citations since 2009 or later, suggesting their importance to their respective fields. Two of the series have over 500 citations.

**Table 1.** Publication status of some of the most contaminated series

GEO series ID	Mycoplasma-mapped RPM	Field of study	Journal	Year of publication	Citations
GSE25183	144 281	Prostate cancer	Nat Biotechnol	2011	271
GSE30772	96 083	Mitochondria biology	Cell	2011	137
GSE45982	84 759	B-cell cancer	Cancer Cell	2013	74
GSE40948	66 905	Embryonic stem cells, chromatin structure	Nat Struct Mol Biol	2012	55
GSE27823	51 752	Enhancers, prostate cancer	Nature	2011	269
GSE49321	36 092	Single cell RNA-seq method	Nature Methods	2013	47
GSE48159	34 902	Estrogen receptor	NA	NA	N/A
GSE50429	34 179	miRNAs, breast cancer	NA	NA	N/A
GSE24447	14 510	Enhancers, developmental biology	Nature	2011	580
GSE45202	13 568	Androgen signaling	Genes Dev	2013	18
GSE36695	12 312	Stem cell biology	Stem Cells Transl Med	2013	2
GSE37003	10 200	RNA modification	Nature	2012	150
GSE48514	10 095	B-cell cancer	Proc Natl Acad Sci	2012	4
GSE43167	9234	microRNA processing	Cell	2013	40
GSE16579	8641	3′UTR, cancer biology	Cell	2009	560
GSE40778	8629	Exon junction complex	Nat Struct Mol Biol	2012	35
GSE15780	8291	Cell survival and apoptosis	Nucleic Acids Res	2011	12
GSE25450	7180	RNA polyadenylation	RNA	2011	115
GSE32340	6573	Viral oncogenes, epigenomics	Genome Research	2012	15
GSE41292	6035	microRNA processing	Cell	2012	45

We looked up the publication information for the indicated series including the journal name, year of publication and the number of citations (according to Google Scholar). N/A denotes the study is not published. Field of study was obtained from analyzing the GEO descriptions and/or paper abstracts.

## DISCUSSION

This study was inspired by a recent incident of mycoplasma contamination in our lab. We wondered how often this occurred in other labs. There have been several such surveys over the last three decades (5–7). These studies used DNA fluorescent staining or polymerase chain reaction-based methods to detect mycoplasma contamination in collected samples. The contamination rates varied from 15 to 70%, although some of the sample sizes were small. Further, a recent analysis of DNA sequences from the 1000 Genomes project suggested 7% of the samples were contaminated (25). This study represents one of the most extensive surveys for mycoplasma contamination. We leveraged publicly available RNA-Seq datasets in GEO to determine the prevalence of contamination today. Importantly, these entries were comprised of various sample types under different experimental conditions and originated from multiple institutions. We estimated that about 11% of GEO series were contaminated based on a cutoff of 100 mycoplasma-mapped RPM in one or more samples. However, this is likely an underestimate. For one, in our analysis, we discarded mycoplasma-mapped reads that were not unique to mycoplasma. Some of these included reads that mapped to other closely related bacteria and hence could not be unambiguously associated with mycoplasma. Secondly, Figure 1 suggests that a cutoff of 10 mycoplasma-mapped RPM was equally powerful to discriminate between cultured and non-cultured samples. At this cutoff, the contamination rate was closer to 15%. Lastly, we found that at least 37% of contaminated series used polyA-selection. Poly(A)-selection should eliminate most host and mycoplasma rRNA. In those series with contamination, it is likely that polyA-selection was inefficient. In our hands it often takes multiple rounds of selection to remove a sufficient amount of rRNA. At such, many of the samples that appeared contamination-free might have simply masked contamination by thoroughly selecting for polyadenylated transcripts. Nonetheless, no matter the exact rate of contamination, our study suggests mycoplasma contamination remains a significant problem.

So what do we do with all these contaminated studies? Should we discard them altogether? Are any of their results valid? These are tough questions that will unfortunately require examination on a case-by-case basis. It should be noted though that mycoplasma contamination does not necessarily invalidate the findings of a study. We can imagine scenarios were certain cellular responses are sufficiently robust even in the presence of such perturbations (if we could call contamination such). Nonetheless, in extreme cases, such as those where mycoplasma RNA comprised more than 10% of all sequence reads, it is hard to imagine the results are not somehow affected. However, we cannot selectively pick which studies we choose to believe. At some point, all contaminated experiments need to be repeated. In the mean time, we suggest GEO and other sequence repositories develop a standard for flagging studies with evidence of mycoplasma contamination. Also, journals should require testing (experimentally and/or computationally if applicable) as a prerequisite for publication.

We mapped sequence reads to four complete mycoplasma genomes: *M. hominis, M. hyorhinis, M. fermentans* and *A. laidlawii*. *M. hominis* and *M. fermentans* are associated with human diseases; the former, pelvic inflammatory disease, the latter, rheumatoid arthritis (26,27). *M. hyorhinis* is commensal in the respiratory tract of pigs (28). *A. laidlawii* has a broad host range but is typically found in cattle. As such, *A. laidlawii* was linked to contamination of commercial bovine serum in the 1970s (7). These four species, along with *M. orale* and *Mycoplasma arginini*, are found in 90–95% of mycoplasma contaminated cell lines (7). In our study, we found *A. laidlawii* was least represented in contaminated samples (Figures 2B-C and 3B-C). This may be indicative of the reduced likelihood of contamination from bovine serum as commercial products are now routinely 0.1 micron sterile-filtered.

We identified 61 genes associated with mycoplasma contamination in a single cell RNA-seq dataset. Unfortunately, we could not identify series containing comparable contaminated and non-contaminated samples. Therefore, we measured the association between the number of mycoplasma-mapped reads per sample and host gene expression in a contaminated series. Consequently, directionality of effect could not be determined. That is, differences in the extent of mycoplasma contamination may have driven host gene expression or differences in host gene expression (especially as it relates to variability of single cells) may have conferred resistance (relatively speaking) to contamination or impacted mycoplasma gene expression. It is also possible that the associations seen here are not biological *per* *se*, but instead indicative of some technical artifacts associated with the presence of the AT-rich mycoplasma transcripts in the sample. Nonetheless, this is equally problematic as it effects the interpretation of the results. Hence, further controlled studies are needed to fully understand the effect of contamination on the quality of RNA-Seq data and possibly how to account for this in data analysis.

Although beyond the immediate scope of this study, we could not help but wonder what interesting aspects of mammalian and mycoplasma biology could be gleamed from this vast, varied and complex nature of the samples comprising these studies. These studies consisted of cell lines of all types, with knockdowns, knockouts and overexpression of many different genes under complex drug treatments. How did the cellular pathways of interest change with the presence of mycoplasma? Does this give us any insight into the biological system? Perhaps more importantly how did these conditions impact the ability of mycoplasma to contaminate cells? After all, mycoplasma is pathogenic in humans and such insight may be helpful in developing therapeutics for diseases such as atypical pneumonia. We hope future studies can tackle some of these questions.

Lastly, our study has broader implications for the analysis of high-throughput sequencing data. Too often in our analysis pipelines, we disregard unmapped reads as technical artifacts or attribute them to problematic or unsequenced regions of the reference genome. While this may be true most of the time, this study suggests such reads warrant further investigation—particularly when they constitute a large number of the total reads. We focused on mycoplasma in this study as a proof of concept due to its known prevalence. However, the methods used here are easily applicable to other known (and unknown) contaminants of cell culture including other bacteria, yeast and viruses.

## SUPPLEMENTARY DATA

Supplementary Data are available at NAR Online.

SUPPLEMENTARY DATA
