# Supplementary material for: Assessing the prevalence of mycoplasma contamination in cell culture via a survey of NCBI's RNA-seq archive
Source: Nucleic Acids Res. 2015 Feb 24;43(5):2535–42. doi: 10.1093/nar/gkv136 (PMC4357728; doi:10.1093/nar/gkv136)
Supplement: SUPPLEMENTARY DATA [file supp_43_5_2535__index.html]

Assessing the prevalence of mycoplasma contamination in cell culture via a survey of NCBI's RNA-seq archive — SUPPLEMENTARY DATA 

# Assessing the prevalence of mycoplasma contamination in cell culture via a survey of NCBI's RNA-seq archive

## SUPPLEMENTARY DATA

**Files in this Data Supplement:**

- SUPPLEMENTARY DATA
